# Clinical Impact of Immunoglobulin Heavy Chain Clonality in Pediatric B‐Cell Precursor Acute Lymphoblastic Leukemia

**DOI:** 10.1002/cam4.71336

**Published:** 2025-11-02

**Authors:** Yuta Katai, Tatsuya Kamitori, Satoshi Saida, Yoshinori Uchihara, Ryo Akazawa, Kiyotaka Isobe, Takashi Mikami, Hirohito Kubota, Itaru Kato, Katsutsugu Umeda, Hiroo Ueno, Junko Takita

**Affiliations:** ^1^ Department of Pediatrics Kitano Hospital, Tazuke Kofukai Medical Research Institute Osaka Japan; ^2^ Department of Hematology/Oncology Saitama Children's Medical Center Saitama Japan; ^3^ Department of Pediatrics, Graduate School of Medicine Kyoto University Kyoto Japan; ^4^ Department of Hematology and Oncology Shizuoka Children's Hospital Shizuoka Japan; ^5^ Department of Pediatric Oncology National Cancer Center Tokyo Japan

**Keywords:** B‐cell lymphoblastic leukemia, clonality, IGH rearrangement, pediatric leukemia, prognosis

## Abstract

**Introduction:**

Recent advancements in risk stratification have greatly improved outcomes in pediatric B‐cell precursor acute lymphoblastic leukemia (BCP‐ALL). Despite favorable prognostic indicators, including the absence of cytogenetic abnormalities and minimal residual disease (MRD) negativity, relapse remains a major clinical concern.

**Methods and Results:**

We investigated the clinical significance of immunoglobulin heavy chain (IGH) clonality using RNA sequencing data in BCP‐ALL. We analyzed IGH clonality from 136 patients. IGH abundance followed a power law distribution, which enabled us to identify disease clones as outliers based on read count. In total, 330 disease clones were detected, and patients were categorized into three clonotype groups: undetectable disease clone (UDC), incomplete disease clone (IDC), and complete disease clone (CDC). Clinical outcomes were compared across clonotypes, including in subgroups with high hyperdiploidy (HHD) and MRD negativity. Among patients with HHD, significant prognostic differences were observed across clonotypes (event‐free survival [EFS], *p* = 0.01; overall survival [OS], *p* = 0.08), even among those who were MRD‐negative (EFS, *p* = 0.01; OS, *p* = 0.03). Furthermore, comparisons of IGH sequences between diagnosis and relapse indicated that while initial disease clones often contributed to relapse, newly expanded clones frequently emerged, particularly in patients with HHD.

**Conclusions:**

These findings highlight the importance of analyzing the IGH repertoire in refining risk stratification and underscore the need for advanced sequencing‐based MRD monitoring.

## Graphical abstract

IGH clonality analysis by RNA sequencing revealed distinct prognostic groups in pediatric BCP‐ALL. Our results demonstrate the value of sequencing‐based IGH profiling to enhance relapse risk assessment and guide precision monitoring strategies.

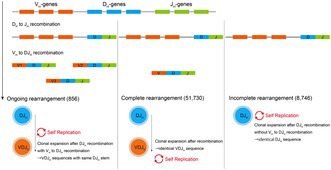

**Abbreviations**
BCP‐ALL: B‐cell precursor acute lymphoblastic leukemia
CDC: complete disease clone
EFS: event‐free survival
FDR: false discovery rate
GSEA: gene set enrichment analysis
HHD: high hyperdiploidy
IDC: incomplete disease clone
IGH: immunoglobulin heavy chain
MRD: minimal residual disease
OS: overall survival
PCR: polymerase chain reaction
RNA‐seq: RNA sequencing
UDC: undetectable disease clone
VDJ: variable, diversity, and joining (segments of immunoglobulin genes)

## 1 Introduction

B‐cell precursor acute lymphoblastic leukemia (BCP‐ALL) is the most common pediatric malignancy [1]. Advances in multiagent chemotherapy regimens and risk‐adapted treatment strategies have improved the survival rates in BCP‐ALL to over 90%. However, relapse occurs in 15%–20% of patients, with an overall survival (OS) of approximately 50% for the relapsed cases [2].

Predictive factors, including clinical features (e.g., age and white blood cell count), response to initial therapy (e.g., prednisolone response and minimal residual disease [MRD]), and leukemic genetic abnormalities, have been established for BCP‐ALL [1, 2, 3]. Among these, aneuploidy and recurrent translocations are important for risk stratification. Notably, *ETV6::RUNX1* (*t*(12;21)(p13;q22)) and high hyperdiploidy (HHD) (51–67 chromosomes) are the most prevalent cytogenetic abnormalities observed in pediatric BCP‐ALL and are associated with a favorable prognosis [4, 5]. However, relapse still occurs in a subset of patients even within these favorable cytogenetic groups, and importantly, some relapses arise in patients who are MRD‐negative following initial therapy [6, 7]. This highlights the critical need for additional prognostic markers capable of identifying high‐risk patients within this otherwise favorable cohort to guide more effective treatment strategies.

**FIGURE 1:**
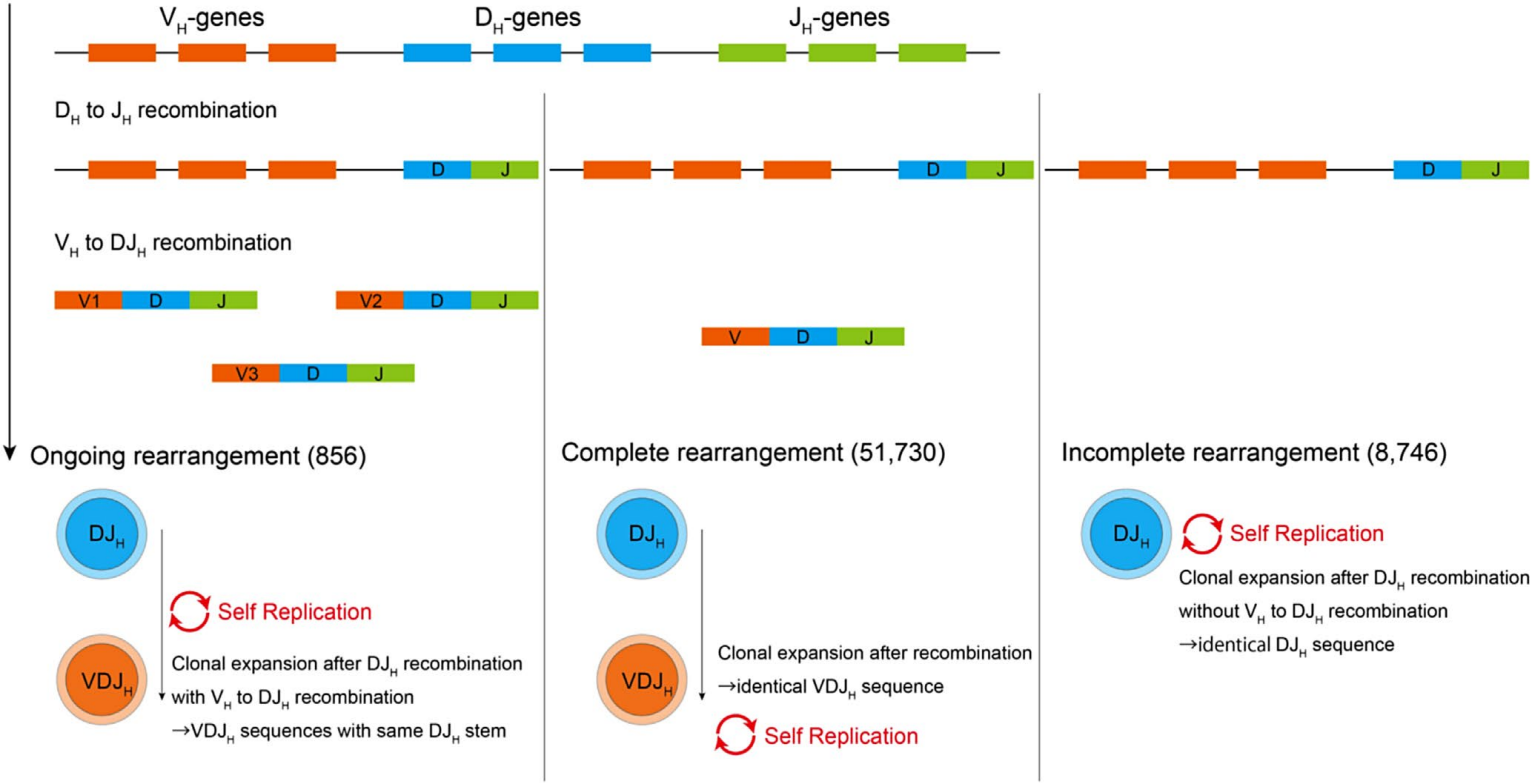
Progression of IGH gene rearrangement: clonal expansion via DJ_H and VDJ_H recombination.

The VDJ (variable [V], diversity [D], and joining [J]) recombination of immunoglobulin heavy chain (IGH) is a hallmark of B‐cell development, which produces unique IGH DNA sequences that serve as molecular fingerprints for MRD monitoring [8, 9]. In the early developmental stages of a pro‐B cell, a D_H_ and a J_H_ gene segment of the IGH locus are joined, which is followed by V_H_ to DJ_H_ recombination. This somatic VDJ_H_ recombination produces a uniquely rearranged IGH DNA sequence at the VDJ_H_ joining region in each B‐lymphocyte. These DNA sequence variations contribute to the overall diversity of antigen receptors and create a distinctive “fingerprint” for leukemia clone‐specific MRD marker design. Disease‐derived IGH clones can be either monoclonal or oligoclonal, and each rearrangement of clones can be categorized into three types: complete V_H_DJ_H_ rearrangement, ongoing V_H_‐DJ_H_ rearrangement presented as multiple V_H_ segments rearranged to the same DJ_H_ stem, and incomplete DJ_H_ rearrangement (Figure 1) [10, 11]. These categorizations may reflect the origin and maturation status of leukemic cells. Although previous studies have investigated the clinical implications of IGH clonality [12, 13], the relationship between IGH clonality and its clinical significance in pediatric BCP‐ALL has not been fully elucidated.

Whole transcriptome RNA sequencing (RNA‐seq) was recently reported as a useful tool for detecting IGH rearrangement transcripts of leukemic disease clones [9]. Although IGH clonality has previously been studied in BCP‐ALL, investigations have mainly relied on DNA‐based methods and have focused primarily on MRD assessment [14, 15], leaving the broader clonal architecture insufficiently explored. In this study, we leveraged RNA‐seq to investigate IGH clonality in pediatric BCP‐ALL, focusing on its clinical relevance across genetic subtypes. By applying a power law model to IGH sequences, we identified prognostic markers, particularly for HHD patients and examined clonal evolution dynamics between diagnosis and relapse.

## 2 Methods

### 2.1 Patients

A total of 136 patients with BCP‐ALL from the TARGET: Pediatric ALL Expansion Phase 2 project, who were enrolled in the Children's Oncology Group clinical trials—P9906 [16], AALL0331 [17], and AALL0232 [18]—were included in this study. RNA‐seq data from diagnostic (*n* = 136) and relapse (*n* = 46) samples were obtained from dbGaP (Study Accession: phs000218.v24.p8) with the approval of the National Cancer Institute Data Access Committee.

### 2.2 Detection of Fusion Genes and Clustering

RNA‐seq reads were counted for each gene using our in‐house pipeline Genomon version 2.6.3 (https://genomon‐project.github.io/GenomonPagesR/). Gene expression levels were normalized, transformed using DESeq2 [19], and clustered. Fusion transcripts were detected using Genomon and Chimerascan [20].

### 2.3 IGH Alignment and Clone Clustering

The RNA‐seq reads were aligned to the V_H_, D, and J_H_ segments of the IGH gene and clustered into clones using MiXCR [21] and Vidjil [22]. MiXCR and Vidjil were used for complete V_H_DJ_H_ and incomplete DJ_H_ rearrangements. Complete V_H_DJ_H_ rearrangements with the same DJ_H_ junction were grouped into one ongoing rearrangement clone if the *p*‐value was < 0.01, based on the Poisson distribution [9]. When clones with complete or ongoing rearrangements between diagnostic and relapse samples were compared, they were assumed to be identical if their DJ_H_ sequences matched.

### 2.4 Detection of IGH Disease Clones Using the Power Law Model

The abundance of IGH followed a power law distribution [23]. Therefore, we estimated the distribution of IGH in each sample using the maximum likelihood method [24]. We then estimated the e‐values by multiplying the total number of IGH clones within the estimated ranges with the probability. The details are described in the Supplementary Method S2.

### 2.5 Estimation of Cell Fractions Using CIBERSORTx

CIBERSORTx [25] was used to estimate the cell fractions from the bulk RNA‐seq data. For this analysis, we first created a signature matrix using a previously published single cell genomic reference map [26] with the “Create Signature Matrix” module. Thereafter, we imputed cell fractions from our bulk expression array using the “Impute Cell Fractions” module.

### 2.6 Gene Set Enrichment Analysis (GSEA)

The Clusterprofiler [27] package was used to implement GSEA. The log_2_ fold changes, calculated using DESeq2, were used as inputs for pre‐ranked GSEA, and *p*‐values were adjusted using the false discovery rate method. Gene sets from Kyoto encyclopedia of genes and genomes [28] and B‐cell lineage were used [29].

### 2.7 Statistical Analysis

Statistical analysis was performed using Python version 3.8.5 for Windows 10 with the Scipy [30], Scikit‐learn [31], and lifelines [32] modules. Unless otherwise specified, the association between categorical variables was tested using the $\chi^{2}$ test. The Mann–Whitney *U* and Kruskal–Wallis tests were used to compare quantitative variables. All statistical tests were two‐sided. In the survival analysis, the log‐rank test was used to compare outcomes between two or more groups, and the survival curve was calculated using the Kaplan–Meier method. Multivariate analysis was based on the Cox regression analysis, and missing values were estimated using multiple imputations.

## 3 Results

### 3.1 Identification of Leukemic Disease Clones Using the Power Law Model

**FIGURE 2:**
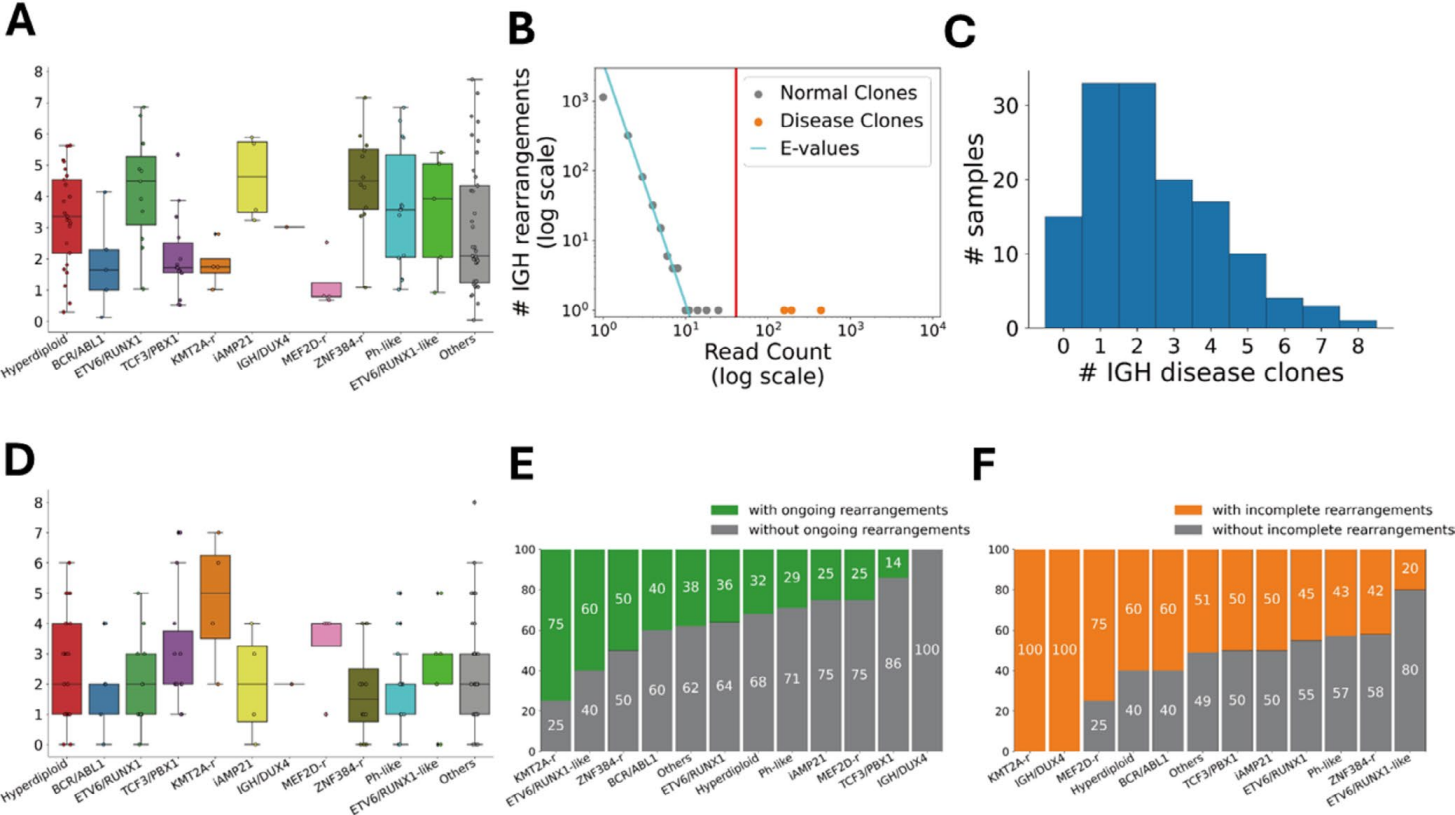
Identification of leukemic disease clones using the power law model. (A) Shannon entropy values were significantly different among the subtypes. (B) Identification of leukemic disease clones using the power law model. Orange dots represent disease clones, gray dots represent normal clones, and the red vertical line represents the threshold. The *x*‐axis is the read count for IGH rearrangement, and the *y*‐axis is number of IGH rearrangements with that read count; both axes are displayed on a log–log scale. (C) Histogram showing the number of disease clones per sample. (D) Number of disease clones by subtype. (E) Percentage of patients with disease clones exhibiting ongoing DJ_H rearrangement, stratified by subtype. (F) Percentage of patients with disease clones exhibiting incomplete rearrangements, stratified by subtype.

We examined the IGH clonality in 136 patients with BCP‐ALL using RNA‐Seq data. The clinical characteristics of the patients are presented in Table 1. *BCR::ABL1*‐like and *ETV6::RUNX1*‐like subtypes were detected via unsupervised clustering using genes with the 500 highest median absolute deviations (Figure S1). We detected 60,476 IGH clones (median, 264; range, 6–4437 per sample; Figure S2)—51,730 with complete VDJ_H_ rearrangements and 8746 with incomplete DJ_H_ rearrangements. Subsequently, we merged 3016 clones with complete rearrangements into 856 clones when multiple V_H_ rearranged to equivalent DJ_H_ sequences harboring ongoing rearrangements (Figure 1). As previously reported, a significant difference was noted in the Shannon entropy [33], a measure of diversity or randomness in a set of sequences, with higher entropy indicating a greater diversity among the genetic subtypes (*p* = 0.002, Figure 2A) [9].

**TABLE 1:** Patient characteristics in the TARGET cohort.

Age at diagnosis, median (range)	6 (1–29)
WBC, median (range)	23,500 (1100–1,148,500)
Gender	
Male	72 (53%)
Female	64 (47%)
MRD day 29	
Positive	52 (60%)
Negative	81 (38%)
Unknown	3 (2%)
Event	
Relapse	101 (78%)
Death from any causes	81 (60%)
Subtype	
Hyperdiploid	25 (18%)
Ph‐like	14 (10%)
TCF3::PBX1	14 (10%)
ZNF384r	12 (9%)
ETV6::RUNX1	11 (8%)
BCR::ABL1	5 (4%)
ETV6::RUNX1‐like	5 (4%)
MEF2Dr	4 (3%)
KMT2Ar	4 (3%)
iAMP21	4 (3%)
IGH::DUX4	1 (1%)
Others	37 (27%)

Abbreviations: MRD, minimal residual disease; WBC, white blood cell.

Subsequently, using the power law model, we identified IGH disease clones with *e*‐values < 0.01 (Figure 2B). Disease clones exhibited significantly high read counts, surpassing the predefined threshold. We detected 330 disease clones (median: 2; range: 0–8 per sample; Figure 2C), comprising 119 complete V_H_DJ_H_ rearrangements, 63 ongoing rearrangements, and 148 incomplete DJ_H_ rearrangements. The number of disease clones per sample was not significantly different among the subtypes (*p* = 0.46), although the *KMT2A* subtype exhibited the highest count, whereas the ZNF384 subtype had the lowest (Figure 2D). Ongoing V_H_‐DJ_H_ rearrangements were most frequent in the *KMT2A*‐rearranged (*KMT2Ar*) (75%), *ETV6::RUNX1*‐like (60%), and *ZNF384*‐rearranged (*ZNF384r*) (50%) subtypes (Figure 2E). Conversely, incomplete DJ_H_ rearrangements were prevalent in the *KMT2A* (100%), *MEF2D*‐rearranged (*MEF2Dr*) (75%), HHD (60%), and *BCR::ABL1* (60%) cases (Figure 2F). These findings indicated that IGH sequence expansion is a characteristic feature of pediatric BCP‐ALL, with subtype‐dependent variability in clonality.

### 3.2 Defining of Three Clonotypes Reflecting the Signatures of Maturity

**FIGURE 3:**
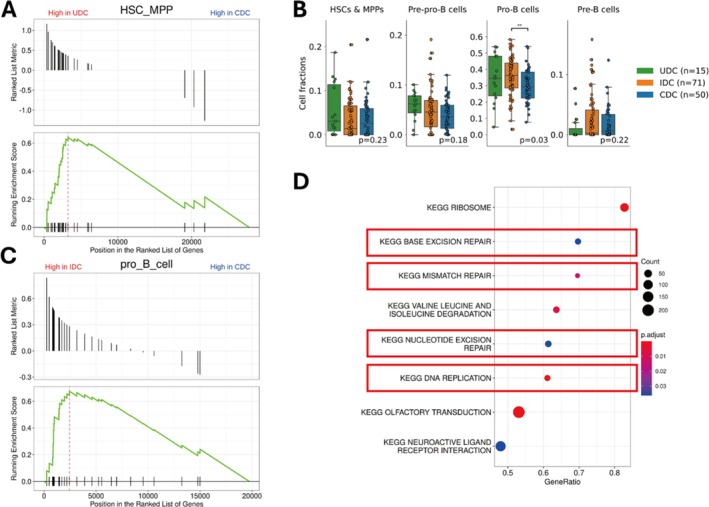
Definition of three clonotypes reflecting maturity signatures. (A) Gene set enrichment analysis (GSEA) showed that genes related to hematopoietic stem cells (HSCs) and multipotent progenitors (MPPs) were significantly upregulated in undetectable disease clone (UDC) patients (FDR < 0.001). (B) CIBERSORTx analysis showed that the median fraction of progenitor cells (HSCs, MPPs, and pre‐pro‐B cells) was the highest in UDC group patients, pro‐B cells in IDC group patients, and pre‐B cells in complete disease clone (CDC) patients. Box plots display the median, first quartile, third quartile, and interquartile range (IQR) with whiskers extending to 1.5 × IQR. (C) Genes related to pro‐B cells were significantly upregulated in incomplete disease clone (IDC) patients (FDR < 0.001). (D) The eight KEGG gene sets with the highest enrichment in GSEA. Gene sets highlighted with red frames are associated with cell cycle progression.

Considering the association between B‐cell maturation status and VDJ recombination steps of IGH, we hypothesized that the IGH clonality of disease clones might reflect the maturation signatures of leukemic cells. Three clonotypes were defined based on the detected disease clones: the undetectable disease clone (UDC) group (*n* = 15), including patients with no disease clones detected; the incomplete disease clone (IDC) group (*n* = 70), including patients with one or more disease clones with incomplete DJ_H_ rearrangements; and the complete disease clones (CDC) group (*n* = 51), which included patients with one or more disease clones with complete V_H_DJ_H_ rearrangements or ongoing V_H_‐DJ_H_ rearrangements, but not those with incomplete DJ_H_ rearrangements. No significant correlation was observed between clonotypes and sex, white blood cell counts at diagnosis, subtypes, or chromosome duplications, which reportedly affect prognosis (Table 2) [34, 35, 36]. The number of disease clones in the IDC group was significantly higher than that in the CDC group (median, 3 vs. 1, *p* < 0.01; Figure S3). To characterize the clonotypes further, we performed GSEA and CIBERSORTx analysis of the RNA‐seq data. GSEA revealed hematopoietic stem cell gene enrichment in UDC group patients compared with that in CDC group patients (FDR < 0.001, Figure 3A). Although hematopoietic stem cell and multipotent progenitor fractions were highest in UDC group patients, the difference was not statistically significant (Figure 3B). Pro‐B cell‐associated genes were significantly upregulated in IDC group patients relative to that in CDC group patients (FDR < 0.001, Figure 3C). In consonance, tumor transcriptomes from IDC group patients were most frequently classified into the pro‐B cell fraction via CIBERSORTx (*p* = 0.03, Figure 3B). Furthermore, cell cycle‐related gene sets were enriched in the IDC group, suggestive of a role in cell proliferation (Figure 3D). Although CDC group patients showed the highest classification into the pre‐B cell fraction, the difference was not statistically significant (*p* = 0.22, Figure 3B). These findings indicated that IGH clonotypes reflect distinct maturation‐like transcriptional states within leukemic cells, with IDC and UDC groups exhibiting more immature B‐cell characteristics.

**TABLE 2:** Association between clonotypes and patient characteristics.

	UDC	IDC	CDC	*p*
	*n* = 15	*n* = 71	*n* = 50	
Age at diagnosis				0.02
< 10	5 (33%)	46 (65%)	36 (72%)	
≥ 10	10 (67%)	25 (35%)	14 (28%)	
WBC				0.74
< 50,000	9 (60%)	46 (65%)	29 (58%)	
≥ 50,000	6 (40%)	25 (35%)	21 (42%)	
Gender				0.34
Male	6 (40%)	36 (51%)	30 (60%)	
Female	9 (60%)	35 (49%)	20 (40%)	
MRD Day 29^a^				0.89
Positive	6 (40%)	28 (39%)	18 (36%)	
Negative	8 (53%)	42 (59%)	31 (62%)	
Subtype				0.84
Hyperdiploid	2 (13%)	15 (21%)	8 (16%)	
Ph‐like	2 (13%)	6 (8.5%)	8 (16%)	
TCF3::PBX1	0 (0.0%)	7 (9.9%)	7 (14%)	
ZNF384r	3 (20%)	5 (7.0%)	4 (8.0%)	
ETV6::RUNX1	1 (6.7%)	5 (7.0%)	5 (10%)	
BCR::ABL1	1 (6.7%)	3 (4.2%)	1 (2.0%)	
ETV6::RUNX1‐like	1 (6.7%)	1 (1.4%)	3 (6.0%)	
MEF2Dr	0 (0.0%)	3 (4.2%)	1 (2.0%)	
KMT2Ar	0 (0.0%)	4 (5.6%)	0 (0.0%)	
iAMP21	1 (6.7%)	2 (2.8%)	1 (2.0%)	
IGH::DUX4	0 (0.0%)	1 (1.4%)	0 (0.0%)	
Others	4 (27%)	19 (27%)	14 (28%)	
Karyotype^b^				
+4	1	8	1	0.21
+10	2	5	4	0.15
+17	1	8	2	0.58
+4 and +10	1	5	1	0.64
+4, +10, and +17	0	4	1	0.57
+17 and +18	1	3	2	0.70
+17 or +18 in the absence of +5 and +20	0	8	2	0.17

Abbreviations: MRD, minimal residual disease; WBC, white blood cell.

^a^ Available in 133 samples (UDC 14, IDC 70, and CDC 49).

^b^ Available in 19 patients with the high hyperdiploid (HHD) subtype.

### 3.3 Survival Analysis Based on IGH Clonotypes in BCP‐ALL

**FIGURE 4:**
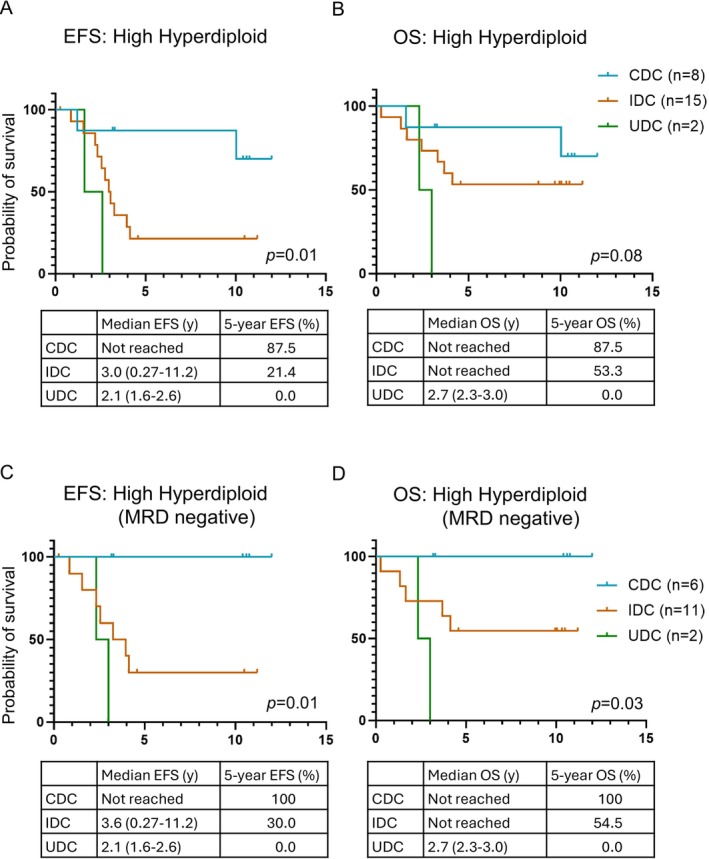
Clinical significance of IGH clonality in BCP‐ALL. (A) Event‐free survival (EFS) differed significantly among the three clonotypes in patients with the HHD subtype (*p* = 0.01). The UDC and IDC clonotypes were associated with poor prognosis. (B) Overall survival (OS) was not significantly different among the three clonotypes (*p* = 0.08); however, UDC and IDC were associated with relatively poorer outcomes. (C) EFS was significantly different among the three clonotypes in patients with the MRD negative HHD subtype (*p* = 0.01). (D) OS differed significantly among the three clonotypes, even in patients with the MRD‐negative HHD subtype (*p* = 0.03).

Survival analyses were performed for the three clonotypes to evaluate their potential as prognostic factors. In the entire cohort, no significant differences in event‐free survival (EFS) or overall survival (OS) were noted among the clonotypes (EFS, *p* = 0.97; OS, *p* = 0.83; Figure S4). Cox regression analysis showed a poor prognosis for the UDC clonotype, although the value was not statistically significant (Tables S1 and S2). However, in the HHD subtype, EFS significantly differed among clonotypes (*p* = 0.01, Figure 4A). The five‐year EFS was lower in the UDC (*n* = 2; 0.0% [95% CI: 0.0%–0.0%]) and IDC (*n* = 15; 21.4% [95% CI: 5.2%–44.8%]) groups compared with that in the CDC group (*n* = 8; 87.5% [95% CI: 38.7%–98.1%]). Similarly, OS was lower in the UDC (0% [95% CI, 0.0%–0.0%]) and IDC (53.3% [95% CI, 26.3%–74.4%]) groups compared with that in the CDC group (87.5% [95% CI, 38.7%–98.1%]), although not statistically significant (*p* = 0.08, Figure 4B). No significant differences in EFS or OS were observed for other genetic subtypes (Figure S4).

Because MRD status is one of the most robust prognostic markers in BCP‐ALL, we assessed its association with IGH clonotypes. Flow cytometry‐based MRD status on day 29 was available for 133 patients. The number of disease clones between MRD‐positive and MRD‐negative patients was not significantly different (*p* = 0.70), and the MRD status was not significantly correlated with any clonotype (*p* = 0.89). Notably, even in MRD‐negative HHD patients, significant differences found in both EFS (*p* = 0.01, Figure 4C) and OS (*p* = 0.03, Figure 4D) were low in the UDC (*n* = 2; OS, 0.0% [95% CI, 0.0%–0.0%]; EFS, 0.0% [95% CI, 0.0%–0.0%]) and IDC (*n* = 11; OS 54.5% [95% CI, 22.9%–78.0%]; EFS 30.0% [95% CI, 7.1%–57.8%]) groups compared with that in the CDC group (*n* = 6; OS, 100.0% [95% CI, 100.0%–100.0%]; EFS, 100.0% [95% CI, 100.0%–100.0%]). Pairwise comparisons of survival among the clonotypes for each panel in Figure 4A–D are provided in Table S3. MRD‐positive HHD patients (*n* = 6) exhibited no significant differences in EFS or OS between the clonotypes (Figure S4).

### 3.4 Analysis of Clonality From Diagnosis to Relapse

**FIGURE 5:**
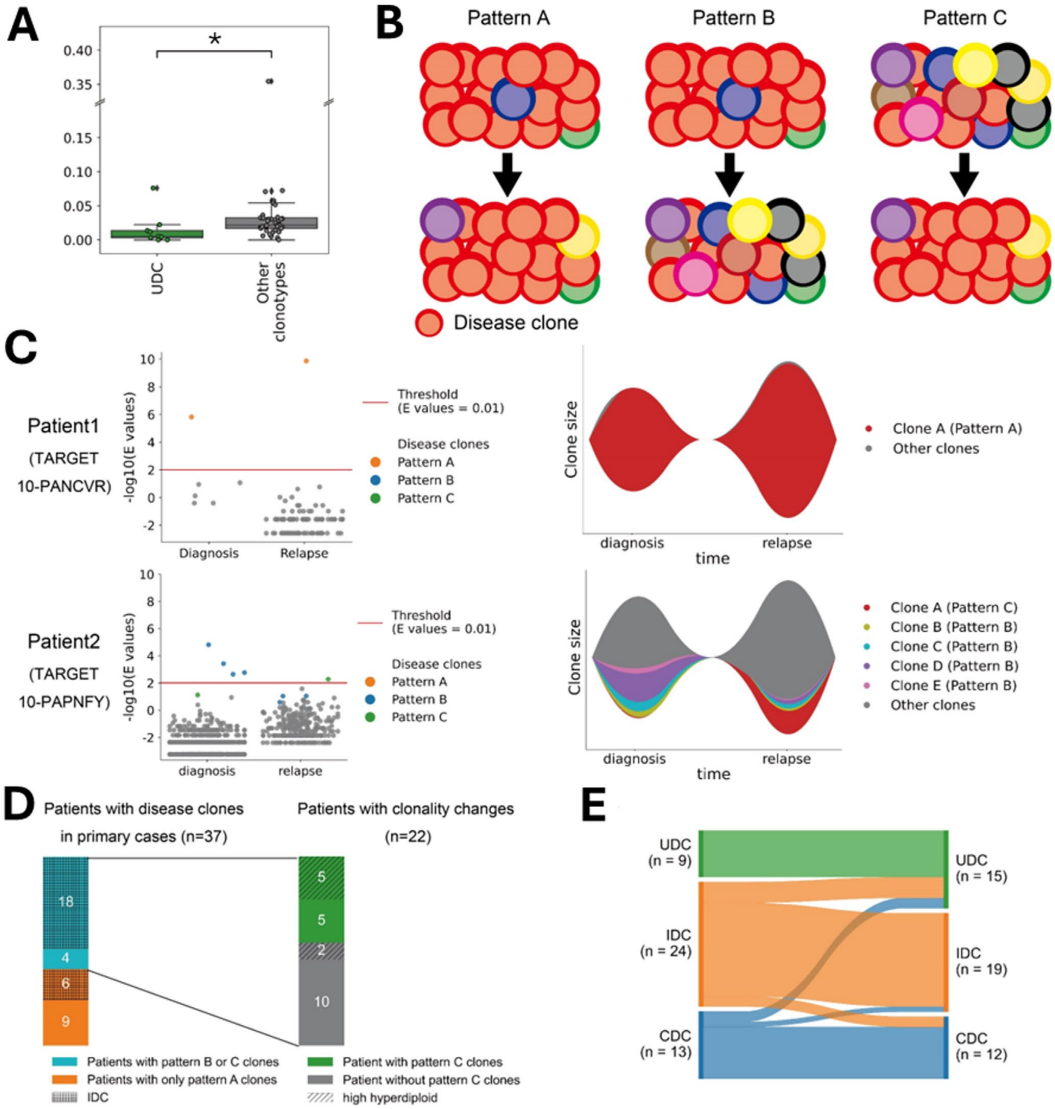
Clonality analysis from diagnosis to relapse. (A) The Morisita–Horn index was significantly lower in patients with the UDC clonotype compared with that in patients with other clonotypes (*p* = 0.03). Asterisks indicate significant differences (*p* < 0.05). (B) Patterns of clonal evolution from diagnosis to relapse. Circles represent disease clones. Pattern A: The major disease clone remains dominant at both diagnosis and relapse. Pattern B: The major clone at diagnosis shrinks at relapse. Pattern C: A previously minor or undetectable clone at diagnosis expands at relapse. (C) Representative data illustrating the IGH clonal evolution. The left panel shows a fish plot representing the IGH clonal evolution from diagnosis to relapse, with window heights corresponding to IGH clone sizes (minor clones are merged into the gray window). The right panel displays e‐values of IGH clones at diagnosis and relapse, where each dot represents an IGH clone with an identical sequence. Patient 1 had only one pattern A clone (upper panel). Patient 2 had four pattern B clones and one pattern C clone (lower panel). (D) Association of clonal evolution patterns with clonotypes and genetic subtypes. The left bar graph shows the association between clonotypes and clonality changes in 37 patients with detectable disease clones at diagnosis. Hatching represents clonotypes at diagnosis, whereas colors indicate types of clonality changes. The bar graph on the right shows the association between the HHD subtype and clonality changes in 22 patients with pattern B or C disease clones. Hatching represents the HHD subtype, whereas colors indicate types of clonality changes. (E) Shift in clonotypes from diagnosis to relapse. Clonotypes remained consistent between diagnosis and relapse in 80% of patients.

Finally, to elucidate the clonal evolution of BCP‐ALL using IGH sequences, IGH clones between diagnostic and relapse samples were compared for 46 patients with relapsed BCP‐ALL. We detected 25,850 IGH clones from samples at diagnosis (median, 346.5; range, 6–2606 per sample; Figure S5A, Table S4), which included 22,742 clones with complete V_H_DJ_H_ rearrangements and 3108 with incomplete DJ_H_ rearrangements. We integrated 1250 of the 22,742 clones with complete V_H_DJ_H_ rearrangements into 425 clones as ongoing rearrangements when multiple V_H_ segments were rearranged into identical DJ_H_ sequences. From samples at relapse, we also detected 14,302 IGH clones (median, 253.5; range, 8–1485; Figure S5B, Table S5), including 12,318 and 1984 clones with complete and incomplete rearrangements, respectively. Additionally, 477 of the 12,318 clones with complete V_H_DJ_H_ rearrangements were integrated into 172 clones with ongoing rearrangements. Comparing the clonotypes, the Morisita–Horn index, which quantifies the similarity between communities [37], was significantly lower in patients with the primary UDC clonotype than in those with the other clonotypes (*p* = 0.03, Figure 5A), indicating that the IGH population was the most discrepant between diagnosis and relapse in patients with the primary UDC clonotype. This finding indicated that patients with the primary UDC clonotype exhibited greater changes in the IGH clonal composition between diagnosis and relapse, highlighting more pronounced clonal evolution.

We identified 100 disease clones at diagnosis and 77 at relapse. Among the disease clones at diagnosis, 66 were also found at relapse. Fourteen clones at diagnosis were also detected at relapse but had e‐values greater than 0.01, indicating that they had become minor clones. On the contrary, the remaining 20 clones at diagnosis were not detected at relapse. By contrast, nine disease clones at relapse were derived from minor clones at diagnosis, with *e*‐values > 0.01, and two clones at relapse were not detected at diagnosis. Thus, we divided the disease clones into three patterns (Figure 5B: pattern A), disease clone at diagnosis that persisted as a disease clone at relapse; pattern B, a disease clone at diagnosis that shrunk its population and became a minor clone or was undetectable at relapse; and pattern C, a minor or undetectable clone at diagnosis that expanded its population or newly emerged to become a disease clone at relapse. Among the 37 primary cases with identifiable disease clones, we detected pattern A disease clones in 28 patients, pattern B in 18 patients, and pattern C in 10 patients. Representative clonal evolution patterns in individual cases are illustrated in Figure 5C. Notably, clonality changes occurred in 22 of the 37 (59%) primary cases with identifiable disease clones, which harbored either pattern B or C clones or both (Figure 5D). These cases were more frequent in patients with primary IDC than in those with CDC (75% vs. 31%, *p* = 0.01). In addition, 5 of the 10 cases with pattern C clones were of the HHD subtype, of which 3 had pattern A clones and 4 had pattern B clones. In contrast, none of the cases with only pattern A clones had an HHD subtype. These results indicated that clonal turnover may actively occur in leukemic cells with HHD.

No statistically significant correlation was noted between patterns of clonality changes and types of IGH rearrangements (*p* = 0.95). Regarding clinical implications, although not statistically significant, the involvement of the central nervous system was more frequent in patients with pattern B or C clones than in those with pattern A clones (*p* = 0.08, Table S6). Next, we compared the clonotypes between diagnosis and relapse. In 37 (80%) patients, the clonotypes were the same, and all nine UDC group patients at diagnosis were also UDC at relapse (Figure 5E). In contrast, clonotypes changed in nine cases: IDC to UDC (*n* = 4), CDC to UDC (*n* = 2), IDC to CDC (*n* = 2), and CDC to IDC (*n* = 1). Survival analysis showed that these clonotype changes did not affect the OS (*p* = 0.72) or relapse free survival (*p* = 0.54). Overall, these data suggest that clonality turnover from diagnosis to relapse occurs in some cases and may be influenced by molecular subtypes. However, it does not appear to affect prognosis in pediatric BCP‐ALL.

## 4 Discussion

This study highlights the clinical significance of IGH clonality in pediatric patients with BCP‐ALL. Using a power law model to estimate extraordinarily expanded IGH sequences, we observed that IGH clonality reflects leukemic cell signatures and serves as a potential prognostic marker. Three clonotypes identified from immune repertoire analysis exhibited predictive potential for relapse in HHD patients. Notably, HHD patients with the UDC or IDC clonotypes had poor prognoses, even among MRD‐negative cases, which indicated that this marker is independent of the previously reported cytogenetic markers. Our findings also underscore the heterogeneous evolution of IGH clones from diagnosis to relapse, highlighting limitations in the currently used MRD techniques that track only the most dominant immune repertoire sequences.

Although up to one‐third of pediatric ALL cases are characterized by a HHD karyotype [38], which is typically associated with favorable prognosis, predicting relapses in this subtype is useful because of the cases with poor prognosis [6]. Previous reports have shown that several cytogenetic aberrations predict the outcome (e.g., triple trisomy +4, +10, +17) [35]; combination aberration (+17 and +18 or +17 or +18 in the absence of +5 and +20) [36], but there is no definitive prognostic factor for HHD BCP‐ALL, especially in MRD‐negative patients.

By tracking IGH sequence variations, we observed diverse inheritance patterns among leukemic cells. Not all clones present at diagnosis persisted at relapse, and vice versa, suggestive of active clonal evolution over time. Two‐thirds of patients exhibited pattern B or C clones, characterized by the expansion of previously undetectable or minor clones and the shrinkage of initially dominant clones. These findings imply that BCP‐ALL comprises heterogeneous cell populations with varying leukemic subclones [39, 40]. Such dynamics highlight the potential limitations of PCR‐based MRD tracking and emphasize the need for more sensitive approaches, such as next‐generation sequencing or RNA‐seq‐based methods, as employed in this study [41, 42].

To detect IGH disease clones, we used the power law model, which allows for a more robust estimation of IGH populations than is possible with the linear model [9]. A previous method based on linear regression [9] was inapplicable to RNA‐seq data with high depth because IGH sequences with low frequencies were difficult to identify [23]. We surmounted this shortcoming by setting lower estimation boundaries, rendering the model more strenuous. By setting lower boundaries, our power law model allowed for better estimation of rare clones than achieved with the linear model (Figure S6). However, the optimal thresholds for dissecting diseased clones need to be determined. In this study, we set an e‐value threshold of 0.01 based on previous studies; however, we found this threshold to be less sensitive, as minor clones with e‐values above 0.01 sometimes contributed to relapse. Adjusting the *e*‐value threshold or using alternative methods based on *p*‐values may enhance the sensitivity.

CIBERSORTx and GSEA provide several cues for determining how clonotypes affect clinical outcomes in pediatric BCP‐ALL. First, the association of these clonotypes and sample immaturity was validated using both methods, which suggested that IDC and UDC clonotypes may represent more immature leukemic cells associated with poorer prognosis, similar to the *ETV6::RUNX1* subtype [43]. In addition, GSEA revealed significant enrichment in cell cycle‐related gene sets, which might indicate that cell cycle progression hampers VDJ recombination [44] and may lead to a poor prognosis.

Our findings on clonal evolution provide clinical insights into BCP‐ALL. The data suggest that chemotherapy‐sensitive clones may dominate at diagnosis, whereas chemotherapy‐resistant clones may exist as minor populations. A subpopulation of leukemic cells exists in BCP‐ALL and IGH rearrangements at relapse can often be traced back to minor clones at diagnosis [41, 42, 45, 46, 47, 48]. Our data on pattern B/C clones also indicate the heterogeneity of leukemic cells and clonal selection of chemotherapy‐resistant clones. Notably, patients with IDC harbored pattern B/C clones more frequently than did patients with CDC. Considering that the IDC clonotype had a more immature signature and harbored a greater number of disease clones, frequent pattern B/C clones may indicate that the disease clones of patients with IDC originate from more immature cells that generate various subclones harboring diverse IGH rearrangements. The high frequency of pattern B/C clones in the HHD subtype may underlie the poorer prognosis associated with IDC, potentially due to its immature characteristics and high heterogeneity.

Our analysis also revealed distinct patterns of IGH clonality across genetic subtypes, with striking features in KMT2Ar BCP‐ALL. The predominance of ongoing and incomplete IGH rearrangements observed in this subtype is in line with previous studies suggesting an origin from an immature progenitor [49]. Consistently, in infant *t*(4;11) ALL, clonotypic antigen receptor rearrangements were shown to be infrequent and often limited to incomplete DJ_H_ rearrangement, further supporting the concept of derivation from an early hematopoietic precursor [50]. Although we attempted to extend such comparisons to other genetic subtypes, assessing differentiation stage‐related differences proved challenging. Nevertheless, our findings in specific subgroups suggest that variability in IGH clonality across subtypes may reflect differences in the developmental stage of leukemic cells, which could, in turn, be linked to clinical behavior and outcome.

Age at diagnosis is a strong prognostic factor in pediatric BCP‐ALL, with patients aged 10 years or more showing inferior outcomes [51]. Notably, in our cohort, older patients harbored the UDC clonotype more frequently, whereas younger patients exhibited the CDC clonotype more often (Table 2). Although the sample size was limited, this distribution suggests that clonotypic immaturity may partly underlie the adverse prognostic effect of older age, whereas more mature clonotypes are predominant in younger patients and may contribute to the favorable outcome.

The relative sensitivity of RNA‐seq–based IGH analysis compared with that of DNA‐based methods warrants consideration. DNA‐based NGS remains the most sensitive and reliable platform for MRD monitoring, as it can capture both productive and non‐productive rearrangements with high sensitivity [14, 15]. Li et al. reported that RNA‐seq identified more IGH disease clones than conventional Sanger sequencing and provided useful clonal information at diagnosis, but it occasionally missed non‐productive clones, whereas DNA‐based IGH‐Seq yielded deeper coverage albeit with a PCR bias [9]. Thus, RNA‐seq and DNA‐based methods provide complementary information: whereas DNA‐based NGS is indispensable for sensitive MRD detection, RNA‐seq offers additional value by simultaneously providing gene expression data and enabling prognostic stratification based on IGH clonotypes, as demonstrated in our study.

A limitation of this study is that IGH analysis based on RNA‐seq may need to be revised using a different technique because it depends on the expression of IGH. RNA‐seq mainly captures productive clones because transcription mostly occurs during productive and complete rearrangements. One study showed that a method based on RNA‐seq ignored some leukemic clones detected via DNA‐seq [52]. Therefore, we might dismiss some unproductive or incomplete rearrangements, especially in patients with UDC. Thus, verification with a DNA‐seq‐based method is warranted. In addition, we analyzed only the TARGET cohort, comprising patients with a prognosis poorer than the currently reported OS rates for pediatric BCP‐ALL. Although our findings indicate that IGH clonotypes can influence outcomes across other subtypes, the sample size per subtype in the TARGET cohort was insufficient for further validation including that using multivariate analysis. Post hoc power analysis demonstrated low statistical power, indicating a high risk of both type I and type II errors. Therefore, these results should be interpreted with caution. Analysis of a single cohort with a relatively poor prognosis may also introduce bias. Nonetheless, analysis of IGH clones using RNA‐seq on larger, diverse cohorts is expected to substantiate our findings and to potentially uncover more complex pathogenic mechanisms in BCP‐ALL.

In conclusion, IGH clonality analysis revealed the heterogeneity of cell signatures in pediatric BCP‐ALL, possibly reflecting distinct characteristics of each leukemic clone. In this study, we identified new prognostic markers for HHD BCP‐ALL, which may refine current risk stratification strategies, although further studies with larger cohorts are necessary. Finally, our comparative analysis of IGH clonal evolution in patients with relapsed BCP‐ALL sheds light on clonal evolution mechanisms, which may inform strategies for managing relapsed BCP‐ALL.

## Author Contributions

**Yuta Katai:** data curation (lead), formal analysis (lead), investigation (lead), methodology (lead), writing – original draft (lead). **Tatsuya Kamitori:** data curation (equal), formal analysis (equal), investigation (equal), writing – original draft (equal), writing – review and editing (equal). **Satoshi Saida:** supervision (equal), writing – original draft (supporting), writing – review and editing (equal). **Yoshinori Uchihara:** data curation (supporting). **Ryo Akazawa:** data curation (supporting). **Kiyotaka Isobe:** data curation (supporting). **Takashi Mikami:** data curation (supporting), writing – review and editing (supporting). **Hirohito Kubota:** data curation (supporting), writing – review and editing (supporting). **Itaru Kato:** data curation (supporting). **Katsutsugu Umeda:** data curation (supporting). **Hiroo Ueno:** conceptualization (lead), data curation (equal), formal analysis (equal). **Junko Takita:** conceptualization (equal), funding acquisition (lead), project administration (lead), supervision (lead), writing – review and editing (supporting).

## Ethics Statement

This study was approved by the Ethics Committee of the Graduate School and Faculty of Medicine at Kyoto University.

## Consent

The authors have nothing to report.

## Conflicts of Interest

The authors declare no conflicts of interest.

## Supporting information


**Table S1:** cam471336‐sup‐0001‐SupplementaryTables.xlsx.

**Data S1:** cam471336‐sup‐0002‐SupportingInformation.docx.

## Data Availability

RNA sequencing data used in this study were obtained from the Therapeutically Applicable Research to Generate Effective Treatments (TARGET) program. Data on diagnostic and relapse samples from patients with BCP‐ALL were accessed via the database of Genotypes and Phenotypes (dbGaP) under Study Accession number phs000218.v24.p8, with approval from the National Cancer Institute Data Access Committee. The data are publicly available at https://www.ncbi.nlm.nih.gov/projects/gap/cgi‐bin/study.cgi?study_id=phs000218.v24.p8.
