# Supplementary material for: Clinical Impact of Immunoglobulin Heavy Chain Clonality in Pediatric B‐Cell Precursor Acute Lymphoblastic Leukemia
Source: Cancer Med. 2025 Nov 2;14(21):e71336. doi: 10.1002/cam4.71336 (PMC12579820; doi:10.1002/cam4.71336)

**Supplementary Method**

**Detection of IGH disease clones using the power-law model**

The abundance of IGH followed a power-law distribution^1^. Therefore, we retained the probability density function $f(x)$ as follows:

|  | $f(x) = \frac{1}{\zeta(\alpha)x^{\alpha}}$ | (1) |
| --- | --- | --- |

where $\alpha$ is a shape parameter, $x$ is the clone’s read count, and $\zeta(\alpha,x_{min})$ is the Riemann zeta function. We estimated $\alpha$ through the maximum likelihood estimation.^2^ Because IGH abundances do not follow the power-law distribution if abundances are low^1^ and MiXCR did not fully recover clones with low abundances^3^, we set lower boundaries of estimation using the log-likelihood test. Specifically, we started with $x_{min} = 1$ and sequentially increased it until the log-likelihood did not improve significantly or $x_{min}$ reached 5. In addition, we set the upper boundaries of the estimation to $x_{max} = 50$ because the estimation is hampered by leukemia IGH sequences with large abundances. We then estimated the e-values by multiplying the total number of IGH clones within the estimated ranges by the probability. The e-values are described as

|  | $g(x) = Nf(x)$ | (2) |
| --- | --- | --- |

where N is the total number of IGH clones within range.

**References**

1. Weinstein JA, Jiang N, Iii RAW, Fisher DS, Quake SR. High-Throughput Sequencing of the Zebrafish Antibody Repertoire. Science. 2009;324(5928):807-10.
2. Bauke H. Parameter estimation for power-law distributions by maximum likelihood methods. The European Physical Journal B. 2007;58(2):167-73.
3. Bolotin DA, Poslavsky S, Davydov AN, Frenkel FE, Fanchi L, Zolotareva OI, et al. Antigen receptor repertoire profiling from RNA-seq data. Nature Biotechnology. 2017;35(10):908-11. doi: 10.1038/nbt.3979.

**Supplementary Figure 1**

Detection of *BCR-ABL1*-like and *ETV6-RUNX1*-like subtypes by unsupervised clustering using the 500 genes with the highest median absolute deviation


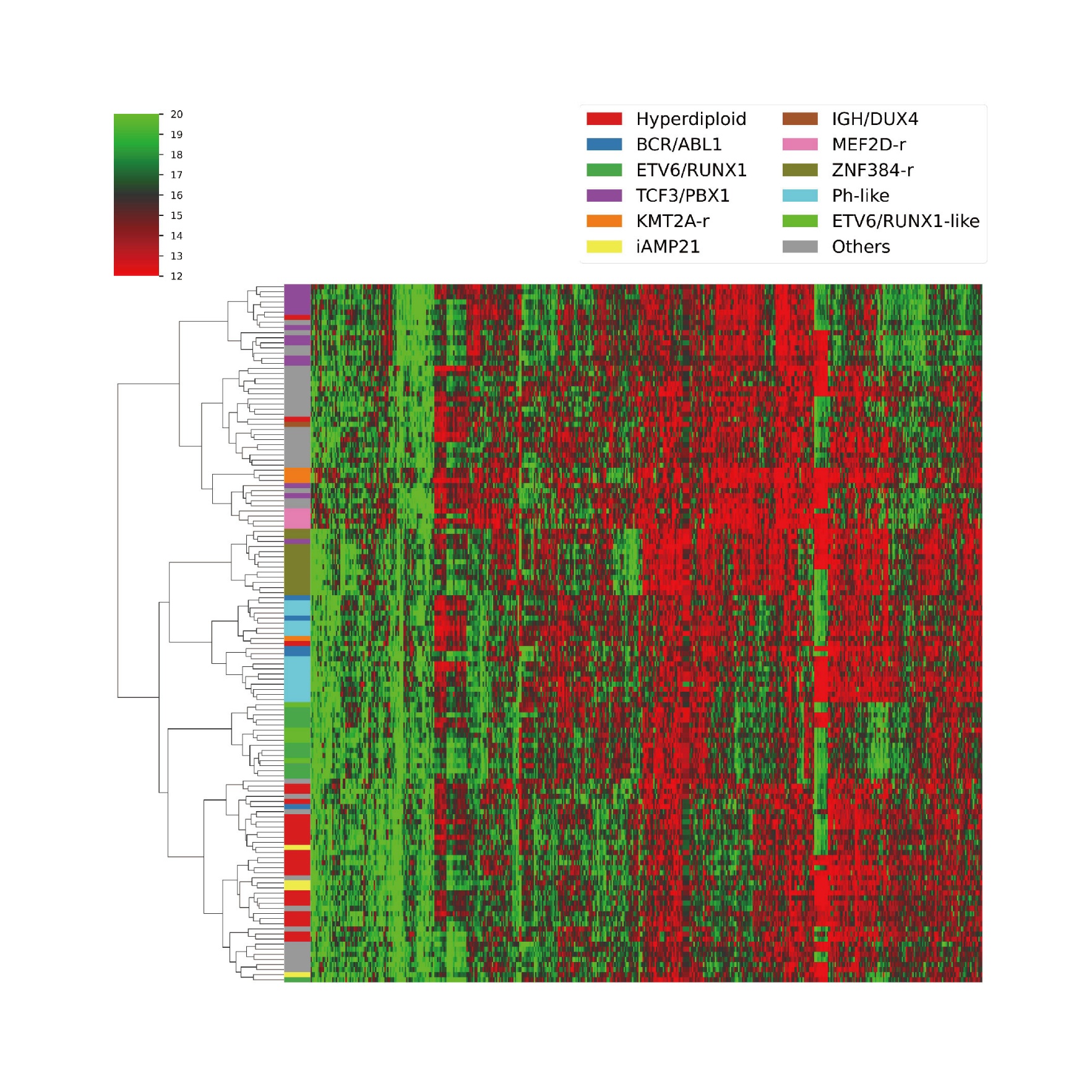


**Supplementary Figure 2**

Number of IGH clones detected per sample.


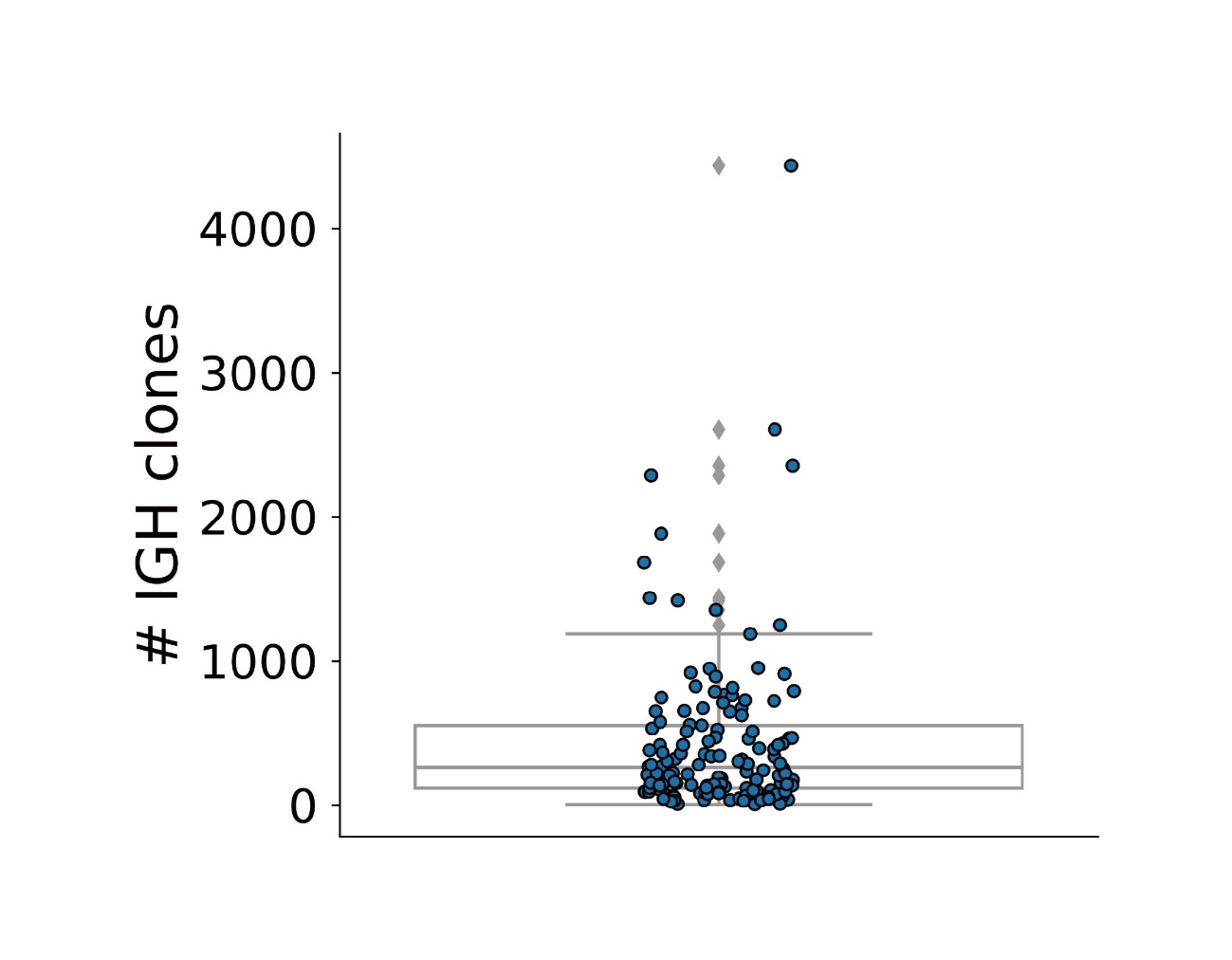


**Supplementary Figure 3**

Comparison of disease clone counts between the IDC and CDC groups.


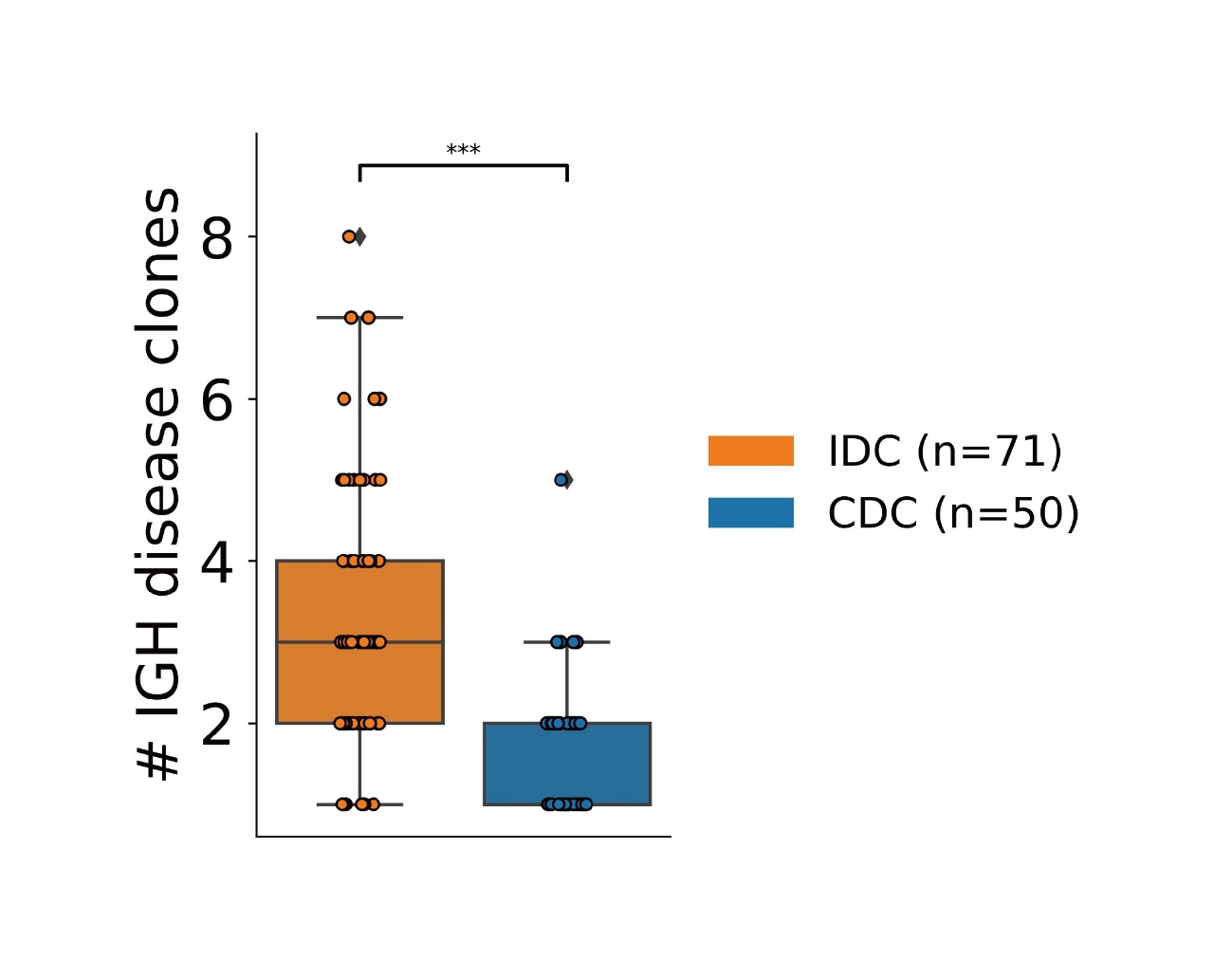


**Supplementary Figure 4**

Survival analysis based on clonotype classification and clonal completeness in pediatric BCP-ALL.

(A) Event-free survival (EFS) did not significantly differ among the three clonotypes in all 136 patients (p = 0.97). (B) Overall survival (OS) did not significantly differ among the three clonotypes (p = 0.83). (C) EFS and OS showed no significant differences among the clonotypes in subtypes other than HHD. (D) EFS did not significantly differ between complete disease clones (CDC) and incomplete disease clones (IDC) in patients with the HHD subtype and positive minimal residual disease (MRD) (p = 0.55). (E) OS did not significantly differ between CDC and IDC in patients with the HHD subtype and positive MRD (p = 0.77).


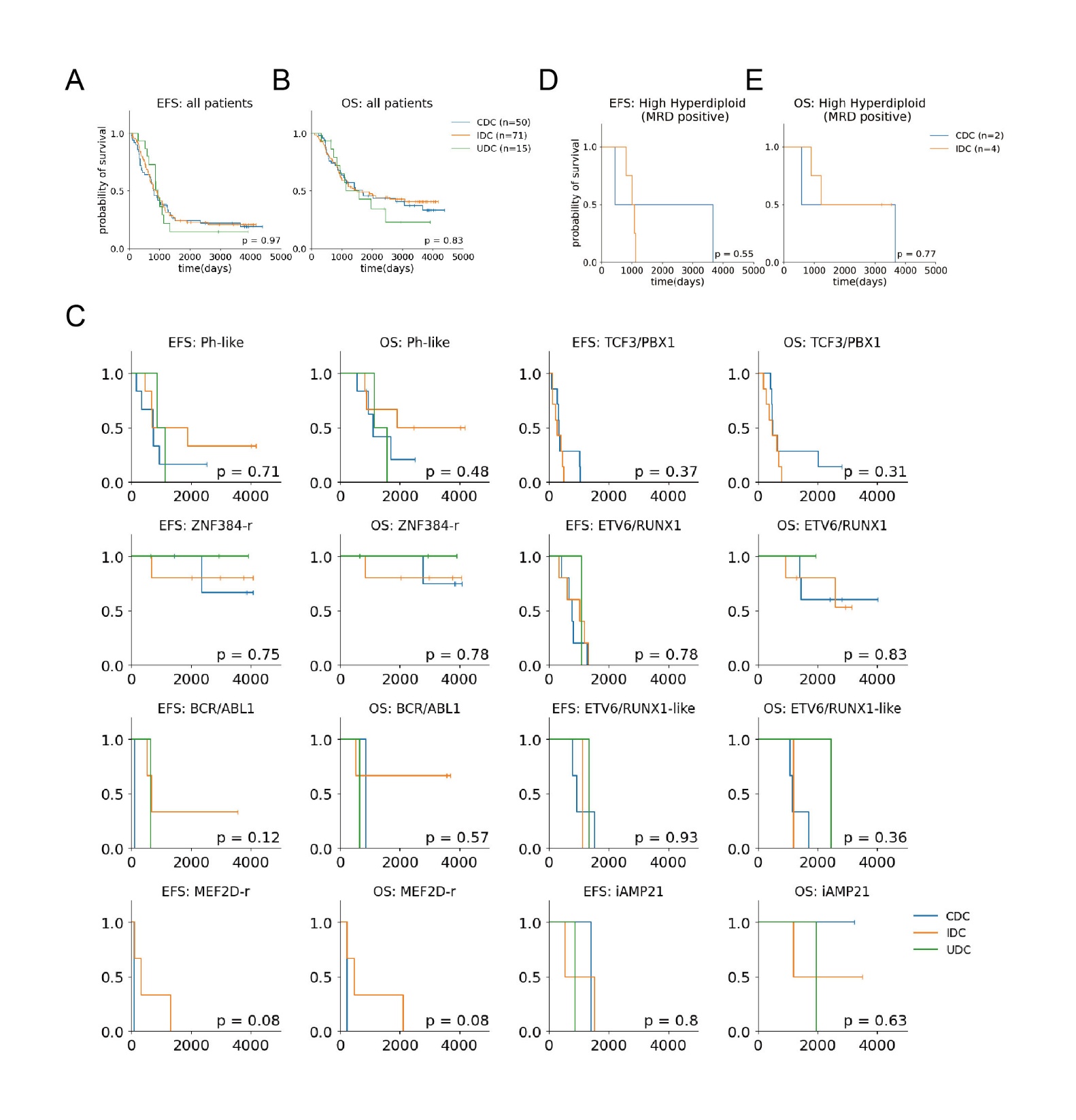


**Supplementary Figure 5**

Number of IGH clones detected per sample at diagnosis (A) and relapse (B).


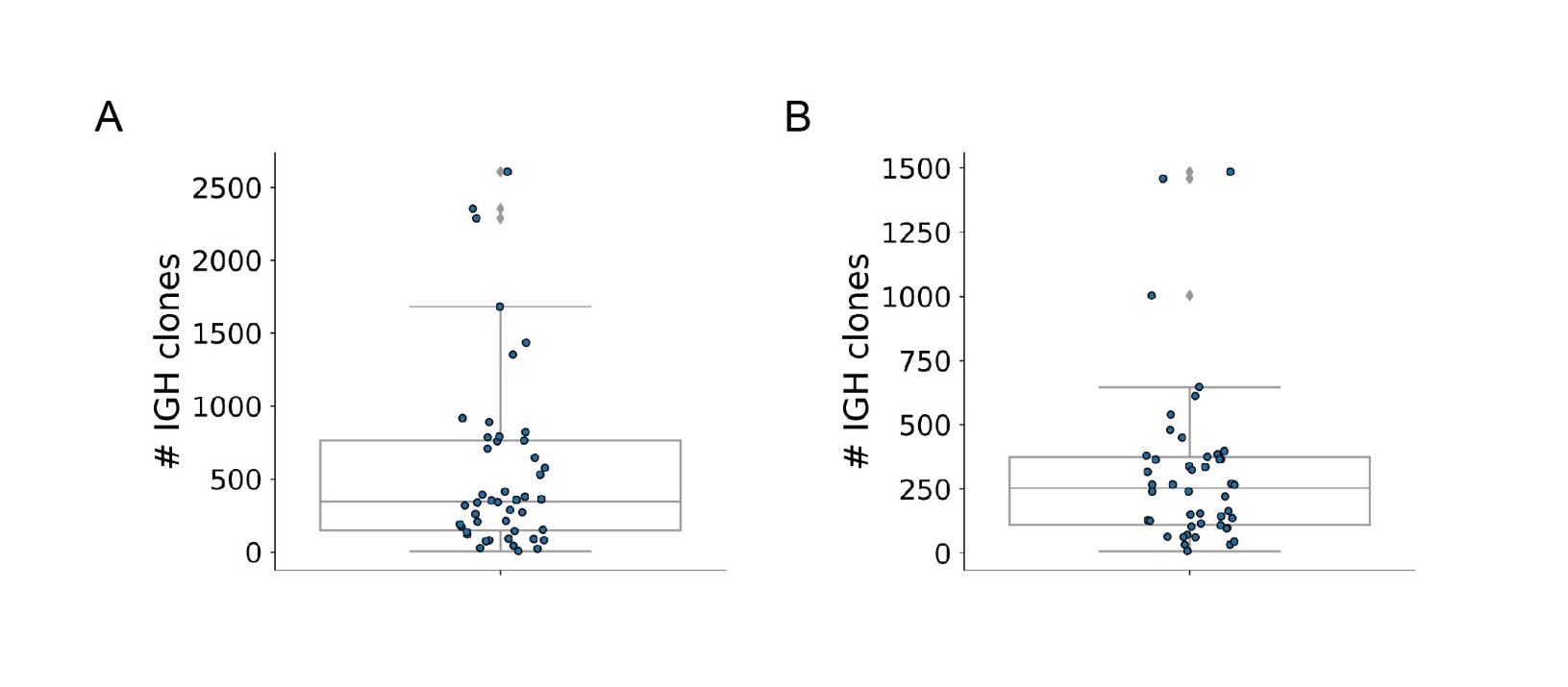


**Supplementary Figure 6**

Differences in e-values between the power-law model and the linear model.


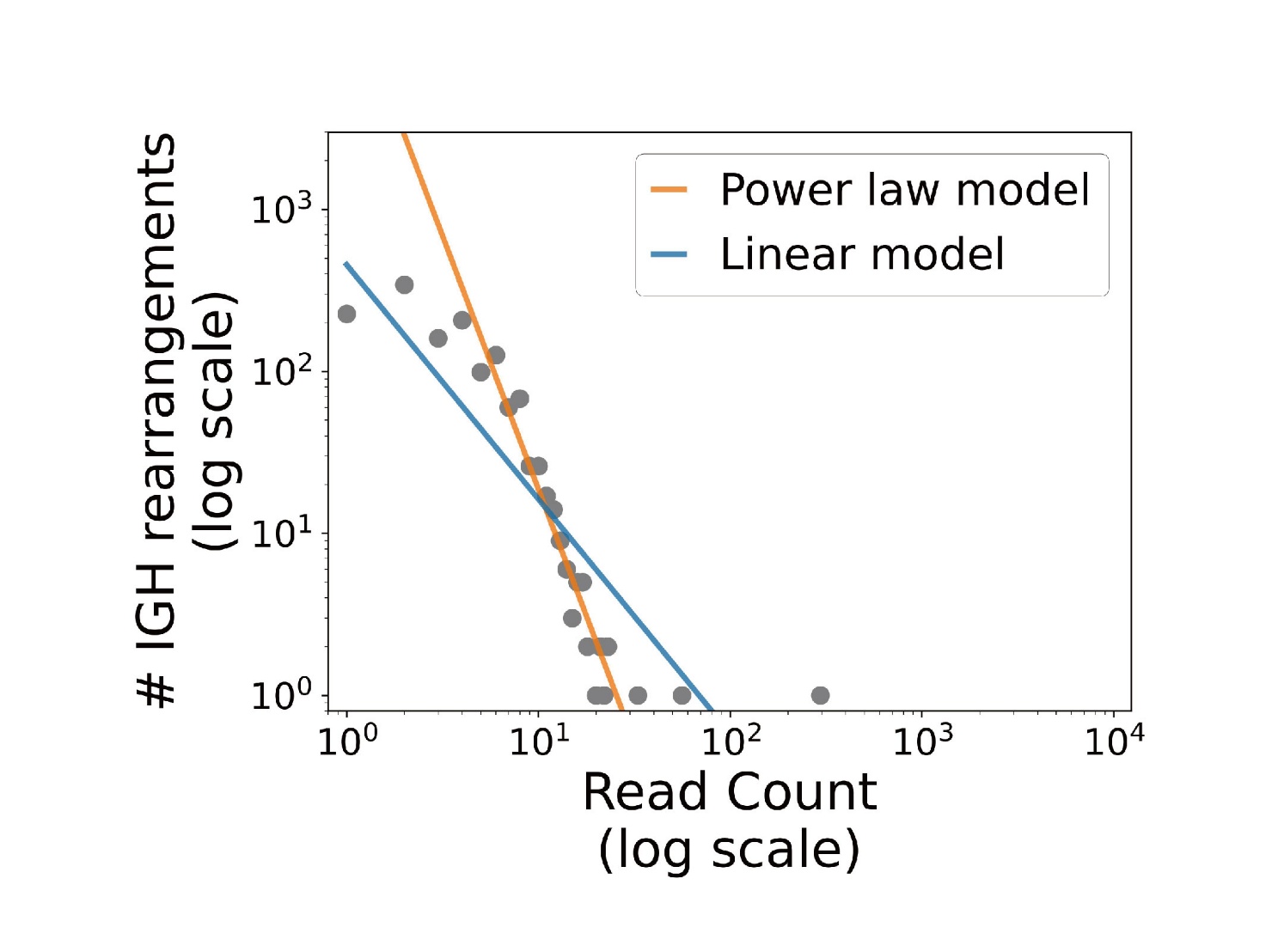

Supplement: Supplementary file 2 — Data S1: cam471336‐sup‐0002‐SupportingInformation.docx. [file CAM4-14-e71336-s002.docx]
